# The *CSRNP* Gene Family Serves as a Prognostic Biomarker in Clear Cell Renal Cell Carcinoma

**DOI:** 10.3389/fonc.2021.620126

**Published:** 2021-03-31

**Authors:** Huaru Zhang, Xiaofu Qiu, Guosheng Yang

**Affiliations:** ^1^The Second School of Clinical Medicine, Southern Medical University, Guangzhou, China; ^2^Department of Urology, Guangdong Second Provincial General Hospital, Guangzhou, China; ^3^Department of Urology, Shanghai East Hospital, Tongji University School of Medicine, Shanghai, China

**Keywords:** clear cell renal cell carcinoma, *CSRNP* family, prognosis, immune infiltration, The Cancer Genome Atlas (TCGA)

## Abstract

The cysteine-serine-rich nuclear protein (*CSRNP*) family has prognostic value for various cancers. However, the association between this proteins and prognosis of clear cell renal cell carcinoma (ccRCC) remains unclear. This study aimed to determine the prognostic value of the *CSRNP* family for patients with ccRCC. Therefore, the gene expression profiling interactive analysis database was used to analyze the mRNA expression of *CSRNP* family members (*CSRNPs*) in relation with survival. Combined and independent prognostic values of CSRNPs were evaluated using SurvExpress and multivariate Cox regression analyses, respectively. Potential signaling pathways impacted by *CSRNPs* were evaluated using Metascape. Associations between the *CSRNP* family and immunocyte infiltration were determined from single-sample gene set enrichment analysis. Both cBioPortal and MethSurv were used to explore whether genomic and epidemic alterations might influence prognosis. We found that when both *CSRNP1* and *CSRNP3* had a low expression, patients with ccRCC had a worse overall survival (OS). Therefore, a prognostic signature was constructed as follows: risk score = −0.224 × exp_mRNA of_ *_CSRNP1_* + 0.820 × exp_mRNA of_ *_CSRNP2_* − 1.428 × exp_mRNA of_ *_CSRNP3_*. We found that OS was worse in patients from the high- than from the low-risk groups (AUC = 0.69). Moreover, this signature was an independent predictor after adjusting for clinical features. Functional enrichment analysis positively associated CSRNPs with the acute inflammatory response and humoral immune response pathways. This was validated by correlating each *CSRNP* with 28 types of immunocytes in tumor and normal tissues. A higher expression of *CSRNP1* and *CSRNP3* was associated with a better prognosis in both the high- and low-mutant burden groups. Cg19538674, cg07772537, and cg07811002 of *CSRNP1*, *CSRNP2*, and *CSRNP3*, respectively, were the predominant DNA methylation sites affecting OS. The *CSRNP* gene family signature may serve as a prognostic biomarker for predicting OS in patients with ccRCC. The association between *CSRNPs* and immune infiltration might offer future clinical treatment options.

## Introduction

Renal cell carcinoma (RCC) has multiple histological subtypes; together, they account for nearly 3% of all human malignant carcinomas (1). The incidence and mortality of RCC continue to increase, and predictions in the United States indicated that 73,750 new cases should be expected in 2020, and that these would directly result in 14,830 deaths (2). The most prevalent (70%–80%) histology of RCC is clear cell renal cell carcinoma (ccRCC) (3). However, 20%–30% of patients with ccRCC have confirmed metastasis at the time of diagnosis (4). Furthermore, although targeted therapy is promising, the 5-year survival rate of patients with metastatic ccRCC remains < 10% (5). Therefore, novel effective biomarkers should be explored to predict the prognoses of patients with ccRCC.

The cysteine-serine-rich nuclear protein (CSRNP) family members, CSRNP1, CSRNP2, and CSRNP3, have been considered as nuclear proteins (6). Their corresponding transcription factors, which are conserved from *Drosophila* to humans (7), play essential roles in many important processes, such as cephalic neural progenitor proliferation, overall zebrafish survival (8), and mouse development (6).

Interleukin-2 induces *CSRNP1* (also known as Axin1 upregulated 1; *AXUD1*) in mouse T cells; it expresses a 1.7 kb transcript with five exons in some malignant cancers, such as kidney, liver, lung, and colon carcinomas (9). Besides, a 4.1 kb *CSRNP2* transcript has been detected in numerous mammalian organs, especially in the brain, ovary, and thymus. Finally, *CSRNP3* (also known as *Mbu-1*) is a brain-specific gene (10); it is expressed in the brain and spinal cords of embryonic to adult mice only (11). These findings suggested that the *CSRNP* gene family might have great value in different cancers. However, few publications have described associations between CSRNP family members and the prognoses of patients with ccRCC.

We therefore explored the distinct expression and multilevel prognostic values of CSRNPs using integrative bioinformatics analysis tools to provide further guidance for the diagnosis and clinical therapy of patients with ccRCC.

## Materials and Methods

### mRNA Expression of *CSRNP*s and Patient Survival

We explored whether the expression of CSRNP family members, which are involved in different clinical stages and affect the prognosis of ccRCC, differed between ccRCC and normal tissues. We therefore analyzed mRNA expression, stage-specific expression, overall survival (OS), and CSRNPs matching normal and genotype-tissue expression (GTEx) data derived from The Cancer Genome Atlas (TCGA), using the Gene Expression Profiling Interactive Analysis (GEPIA) online tool (12) (http://gepia.cancer-pku.cn/), to investigate genomic functionality. We obtained the expression profile of TCGA-KIRC from UCSC Xena (https://xenabrowser.net/datapages/?cohort=GDC%20TCGA%20Kidney%20Clear%20Cell%20Carcinoma%20(KIRC)&removeHub=https%3A%2F%2Fxena.treehouse.gi.ucsc.edu%3A443) and the expression profile and clinical features of GSE29609 from the Gene Expression Omnibus (http://www.ncbi.nlm.nih.gov/geo/).

### Prognostic Values of the CSRNP Family Signature

We aimed to construct a comprehensive CSRNP family signature to better predict the OS of ccRCC patients. The SurvExpress online tool (13) (http://bioinformatica.mty.itesm.mx:8080/Biomatec/SurvivaX.jsp) was utilized to construct and evaluate the prognostic value of the CSRNP family signature. Here, a risk score formula was obtained, and the risk score for each patient was automatically generated. Patients were assigned to high- or low-risk groups based on the median cutoff value of the risk scores. Moreover, the independent prognostic value of the CSRNP family signature was determined using multivariate Cox regression analysis incorporating age, gender, grade, stage, and the signature.

### Functional Enrichment Analysis of Differentially Expressed Genes (DEGs) Between Healthy and Tumor Groups and High- and Low- Risk Groups

We then investigated the correlations between potentially critical pathways and the risk score model. First, DEGs between normal or adjacent tissues and ccRCC (|log_2_FC| > 1 and *P* < 0.05) were detected using volcano plots. Then, samples were classified as belonging to the high- or low-risk groups based on the median cutoff of the risk score model; DEGs between these two groups (|log_2_FC| > 1 and *P* < 0.05) were also identified *via* volcano plots. Finally, DEGs that merged in Venn diagrams were considered as risk-related DEGs and selected for further analysis by Metascape (14) (http://metascape.org/gp/index.html).

### Correlations Between *CSRNPs* and Immune Infiltration

According to the results of the functional enrichment analysis, CSRNP family members may play a role in ccRCC immunotherapy-related signaling pathways. To further verify this finding, we calculated the immune infiltration of 28 immunocytes using a set of genes determined by single-sample gene set enrichment analysis (ssGSEA) (15). Subsequently, the correlations between each *CSRNP* family gene and the 28 immunocytes were evaluated in normal kidney and ccRCC tumor samples.

### Prognosis of Genetic and Epigenetic Changes in *CSRNP* Family Members

Since the transcriptional gene expression profile could be affected by genetic and epigenetic changes (16, 17), we examined whether CSRNP family genetically and epigenetically influenced the prognosis of ccRCC.

First, genetic alterations, which mainly comprised missense and truncating mutations, amplification, and deep deletion, were analyzed using cBioPortal (18) (http://www.cbioportal.org/). Patients were assigned to groups with a high- or a low-mutant burden based on the median cutoff value, and their survival was analyzed using Kaplan-Meier (K-M) curves according to the genetic alterations found in each *CSRNP* family gene.

Then, we assessed epigenetic changes in the *CSRNP* family, and evaluated the relative DNA methylation site data from TCGA using the comprehensive bioinformatics platform MethSurv (19) (https://biit.cs.ut.ee/methsurv/). Moreover, the prognostic values of all methylation sites associated with CSRNP family members were assessed.

### Statistical Analysis

Univariate and multivariate Cox regression analyses of CSRNP family members were performed for assessing the OS of patients, using hazard ratios (HR) and a 95% confidence interval (95% CI). Paired t-tests were conducted to compare tumor and adjacent normal tissues from patients from the TCGA-KIRC dataset. OS was evaluated using the K-M curves. *P* values < 0.05 were considered statistically significant.

## Results

### mRNA Expression Levels of *CSRNP* Family and OS

**Figure 1:**
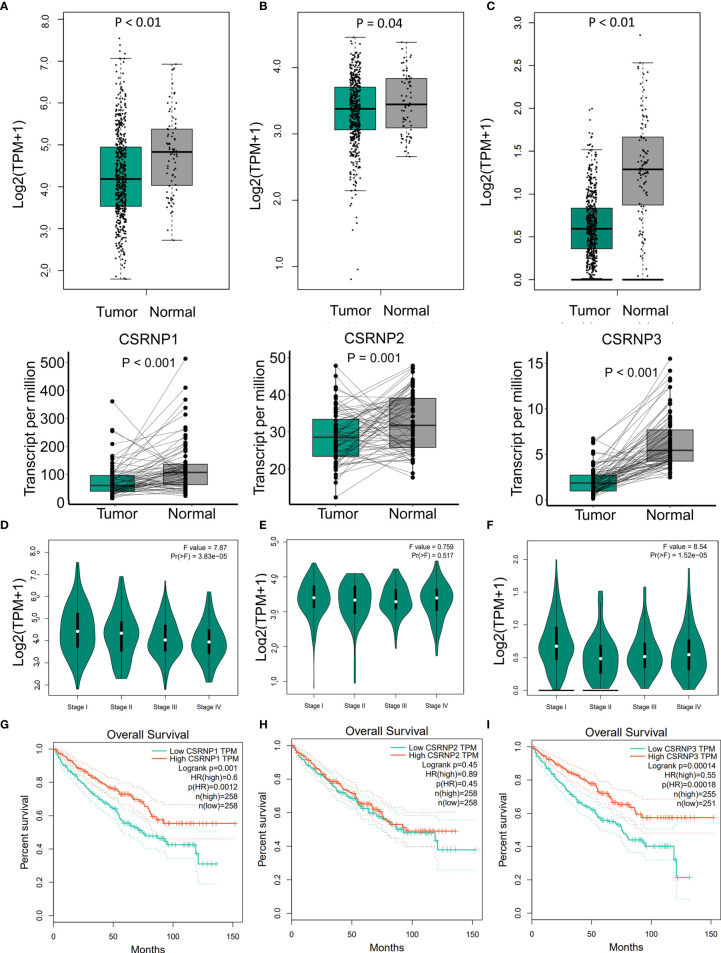
mRNA expression, stage-specific expression, and overall survival of CSRNP gene family members according to GEPIA. **(A–C)** mRNA expression of CSRNP family members in tumor and normal tissues (upper image), and paired tumor and normal tissues (lower image). Green: tumor tissues; gray: normal tissues. **(D–F)** Stage-specific mRNA expression of CSRNP family members. **(G–I)** Kaplan-Meier curves of the overall survival analysis in relation to the CSRNP family.

*CSRNP1*, *CSRNP2*, and *CSRNP3* were significantly less abundant in ccRCC (n = 532) than in normal (n = 72) tissue sample data from TCGA-KIRC database (**Figures 1A–C**). Moreover, comparisons of paired tumor and adjacent normal tissues from patients generated similar results (**Figures 1A–C**). We also found that *CSRNP1* displayed significantly different stage-specific expression: the more advanced the ccRCC stage, the lower the *CSRNP1* expression (**Figure 1D**). However, the expression of *CSRNP2* did not differ between stages (**Figure 1E**), whereas *CSRNP3* showed a higher expression in stage I than in stage II-IV ccRCC samples (**Figure 1F**). We also compared the expression of *CSRNP*s across different Fuhrman grades based on GSE29609 and found no significant differences (**Figure S1**).

We then evaluated whether *CSRNP* mRNA levels affected the prognosis of ccRCC, and found that high mRNA levels of *CSRNP1* (HR: 0.60, *P* = 0.001) and *CSRNP3* (HR: 0.55, *P* < 0.001) were significantly correlated with favorable OS (**Figures 1G, I**). In contrast, the mRNA expression of *CSRNP2* was not significantly associated with a favorable OS (**Figure 1H**).

### Combined Prognostic Value of the *CSRNP* Family Signature

**Figure 2:**
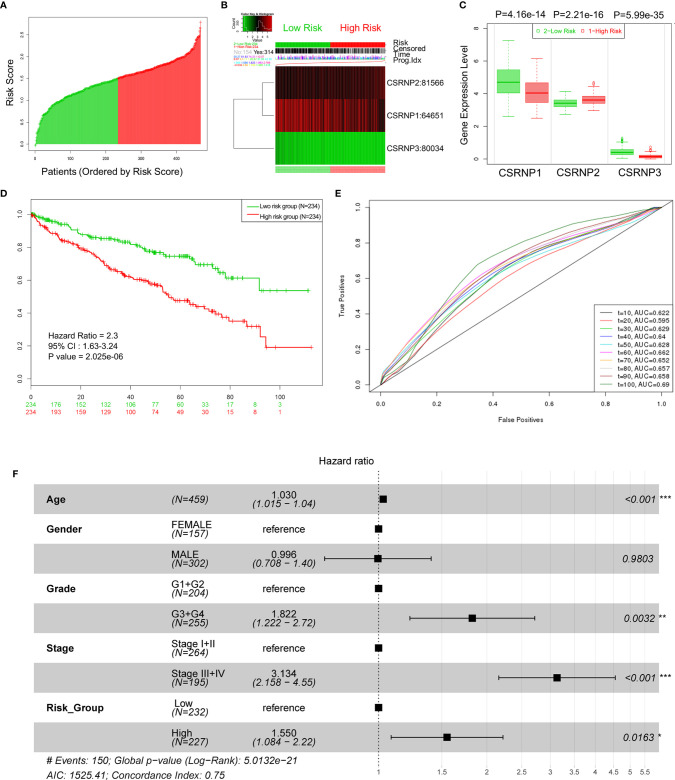
Prognostic values of the CSRNP family signature determined using SurvExpress. **(A)** Patients were assigned to high- and low-risk groups based on median cutoff risk scores. **(B)** Heat map of CSRNP family members expression. **(C)** Comparison of the expression of *CSRNP* genes between low- and high-risk groups. **(D)** Survival analysis of low- (green) and high-risk (red) groups. **(E)** Time-dependent receiver operating characteristics (ROC) curves. **(F)** Multivariate Cox regression analysis of variables and CSRNP family signature risk scores. *P < 0.05, **P < 0.01, ***P < 0.001.

We constructed a CSRNP family signature risk score model as follows: risk score = −0.224 × exp_mRNA of_ *_CSRNP1_* + 0.820 × exp_mRNA of_ *_CSRNP2_* – 1.428 × exp_mRNA of_ *_CSRNP3_*, according to the coefficient indexes shown in **Table 1**. Differences in the expression patterns of CSRNPs were observed between the low- and high-risk (n = 234 each) groups based on the median cutoff value of risk scores. A lower expression of *CSRNP1* and *CSRNP3* was observed in the high-risk group, whereas there was a higher expression of *CSRNP2*, compared to those levels observed in the low-risk group (**Figures 2A–C**). As expected, the low-risk group had a better OS than the high-risk group (**Figure 2D**; HR: 2.30, 95% CI: 1.63–3.24, *P* < 0.001). Moreover, the area under the curve (AUC) of a time-dependent ROC increased to 0.69 during the follow-up period (**Figure 2E**). In addition, we compared the distribution of clinical features between the low- and high-risk groups and found similar age and sex distributions between them. However, more patients in the high-risk group had advanced tumor stages or tumor grades (**Table 2**).

**Table 1:** Cox proportional hazard regression analysis result shows the coefficient of CSRNP family.

	Co-ef	Exp(coef)	Se(coef)	Z	Pr>|Z|
CSRNP1	-0.224	0.799	0.092	-2.429	0.01513
CSRNP2	0.820	2.271	0.255	3.213	0.00131
CSRNP3	-1.428	0.240	0.442	-3.228	0.00125

Co-ef, co-efficient; Exp (co-ef), Expectation (co-ef); Se (co-ef), standard error (coef).

**Table 2:** Summarization of clinical features.

	Subgroup	Low risk	High risk	*P*
Age (years)		60.59 ± 12.10	60.60 ± 12.29	0.994
Gender (%)	Female	82 (35.3)	75 (33.0)	0.673
	Male	150 (64.7)	152 (67.0)	
Grade (%)	G1+G2	128 (55.2)	76 (33.5)	<0.001
	G3+G4	104 (44.8)	151 (66.5)	
Stage (%)	Stage I+II	162 (69.8)	102 (44.9)	<0.001
	Stage III+IV	70 (30.2)	125 (55.1)	

Results from a multivariate Cox regression analysis suggested that the CSRNP family signature was an independent predictor for the prognosis of patients with ccRCC (**Figure 2F**; HR: 1.550, 95% CI: 1.084–2.220, *P* = 0.0163).

### Functional Enrichment Analysis of *CSRNP* Impacted Genes

**Figure 3:**
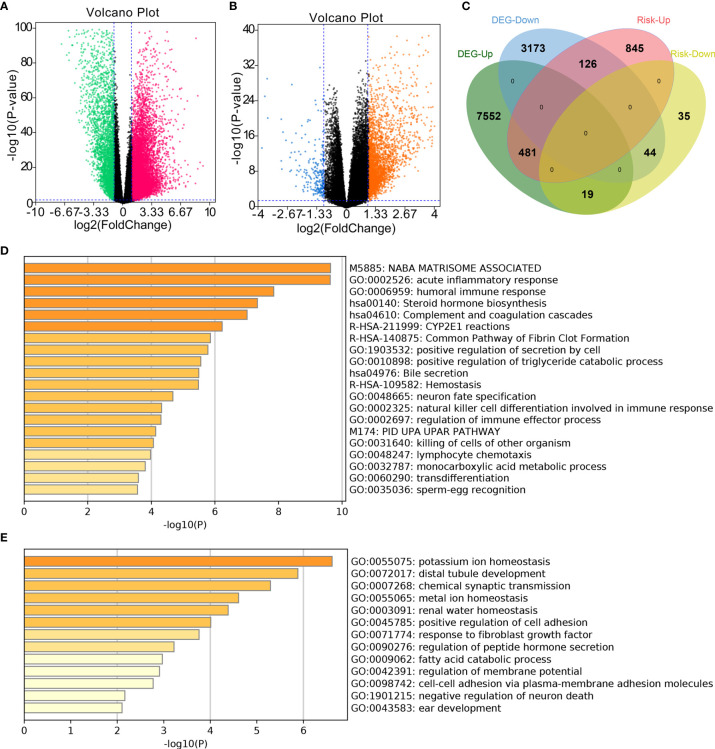
Functional enrichment analysis of differentially expressed genes (DEGs). **(A)** Volcano plot of DEGs between patients with and without ccRCC. **(B)** Volcano plot of DEGs between patients in the high- and low-risk groups. **(C)** Venn diagram merging DEGs from **(A, B)**. **(D, E)** Functional enrichment analysis of significantly **(D)** upregulated genes associated with both tumors and increased risk of tumors, and **(E)** downregulated genes associated with tumors and decreased risk of tumors.

Significant DEGs between normal and ccRCC tumor tissues (| log_2_FC| > 1 and *P* < 0.05; **Figure 3A**) and between high-and low-risk groups were selected (|log_2_FC| > 1 and *P* < 0.05; **Figure 3B**). Then, DEGs that were significantly upregulated in tumor tissues and the high-risk group (481 genes) and those significantly downregulated in tumor tissues and the low-risk group (44 genes) (**Figure 3C**) were further analyzed using Metascape. The results showed that CSRNPs were associated with different pathways, including the acute inflammatory response, humoral immune response, natural killer cell differentiation involved in immune response, and regulation of immune effector process (**Figures 3D, E**).

### Correlations Between CSRNPs and Immune Infiltration

**Figure 4:**
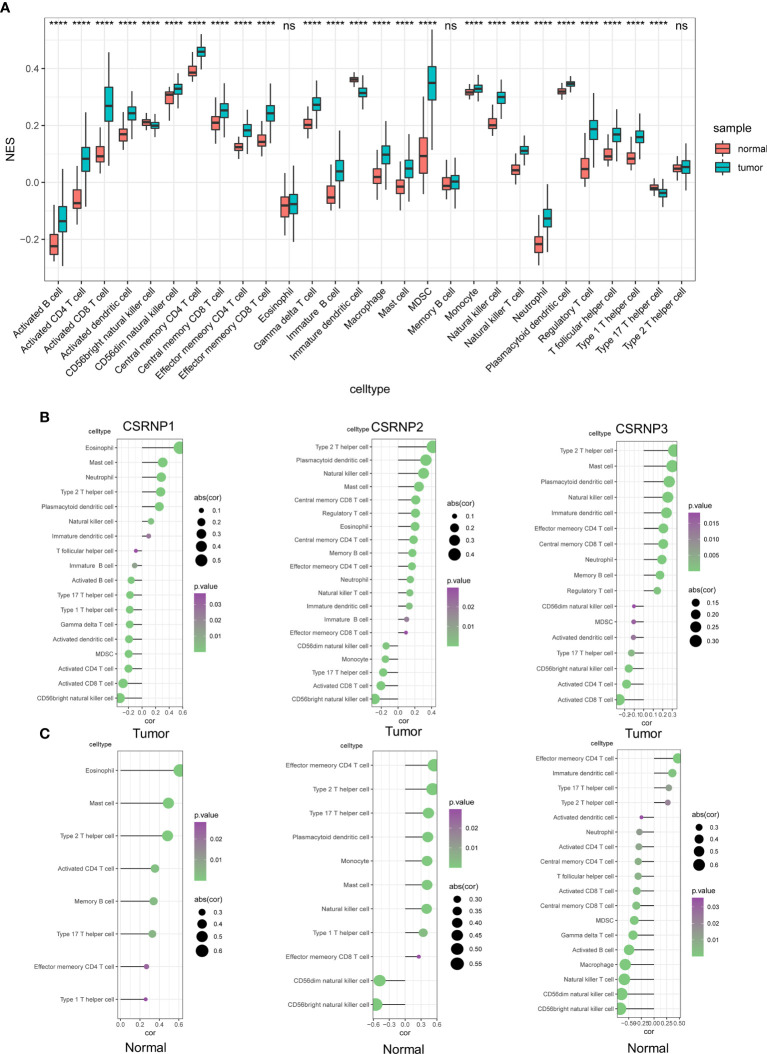
Correlations between CSRNP family and immune infiltration. **(A)** Normalized enrichment scores (NES) of 28 immunocytes between normal and tumor tissues. **(B, C)** Correlations between CSRNP family and significant infiltrated immunocytes in tumor **(B)** and normal **(C)** tissues.

The functional enrichment analysis associated the CSRNP family with immune infiltration signaling pathways. To further verify this, we calculated the immune infiltration of 28 immunocytes between tumor and paracancerous tissues, and found that 22 out of the 28 immunocytes were more abundant in tumor than in paracancerous tissues (**Figure 4A**).

We then evaluated the correlation between *CSRNP*s and the 28 immunocytes in normal kidney and ccRCC samples. The results indicated that the immune infiltration profiles of the *CSRNP*s differed between normal and ccRCC tissues. Moreover, the CSRNP family was significantly correlated with more immunocytes in tumor than in normal tissues. We found that all three *CSRNP*s were positively associated with the infiltration of type 2 T helper cells, mast cells, and natural killer cells, and negatively associated with the abundance of CD56^bright^ natural killer cells and activated CD8 T cells (**Figure 4B**). These results indicated that the CSRNP family might impact the immune environment of ccRCC through the above-mentioned immunocytes. The immune infiltration profile is different in normal kidney tissues. CSRNP1 only positively regulated the infiltration of eight immunocytes and did not negatively regulate any, which was significantly different from its effects in ccRCC tissues. Meanwhile, CSRNP2 and CSRNP3 were both mostly positively associated with effector memory CD4 T cells, but not with type 2 T cells in tumor tissues (**Figure 4C**).

### Genetic Alteration in *CSRNP*s

**Figure 5:**
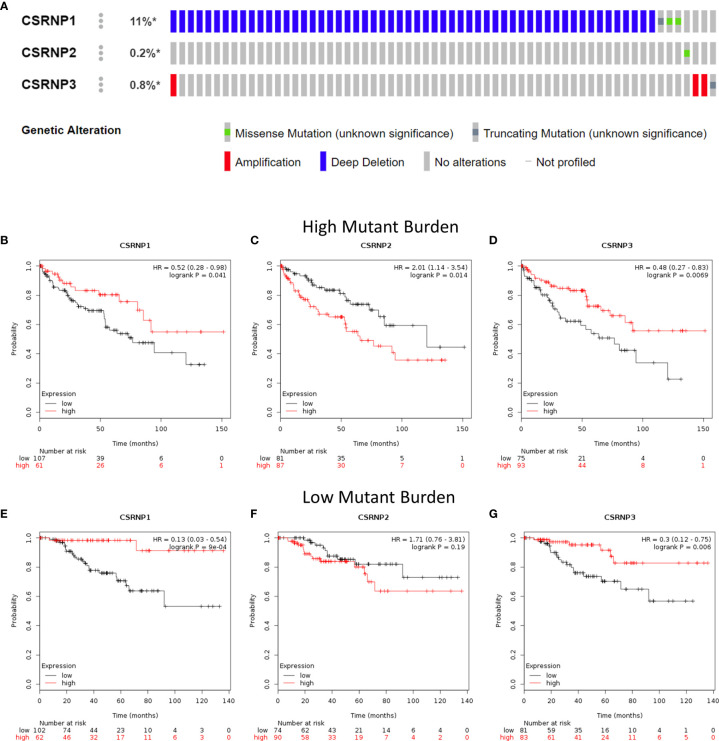
Genetic alterations and overall survival in patients with high- and low-mutant burden within CSRNP family according to cBioPortal. **(A)** Genetic alterations of *CSRNP* genes in patients from TCGA dataset (each rectangle represents one patient; not all patients were shown [n = 532]); **(B–G)** Overall survival of patients with high- **(B–D)** and low- **(E–G)** mutant burden within CSRNP family.

Genetic alterations play an important role in the regulation of gene expression. We found that the genetic alterations in *CSRNP1*, *CSRNP2*, and *CSRNP3* were approximately 11%, 0.2%, and 0.8%, respectively (**Figure 5A**). We then evaluated the prognostic values of *CSRNP*s in high- and low- mutant burden patients in all enrolled patients with ccRCC, and found that both *CSRNP1* and *CSRNP3* act as protective factors in both high- and low-mutant burden patients (**Figures 5B, E, D, G**). Meanwhile, although we did not find a significant effect of *CSRNP2* on ccRCC OS in the entire group, we found that *CSRNP2* was a remarkable hazard factor in patients with a high-, but not with a low-mutant burden (**Figures 5C, F**).

### DNA Methylation Sites Within *CSRNP*s

**Figure 6:**
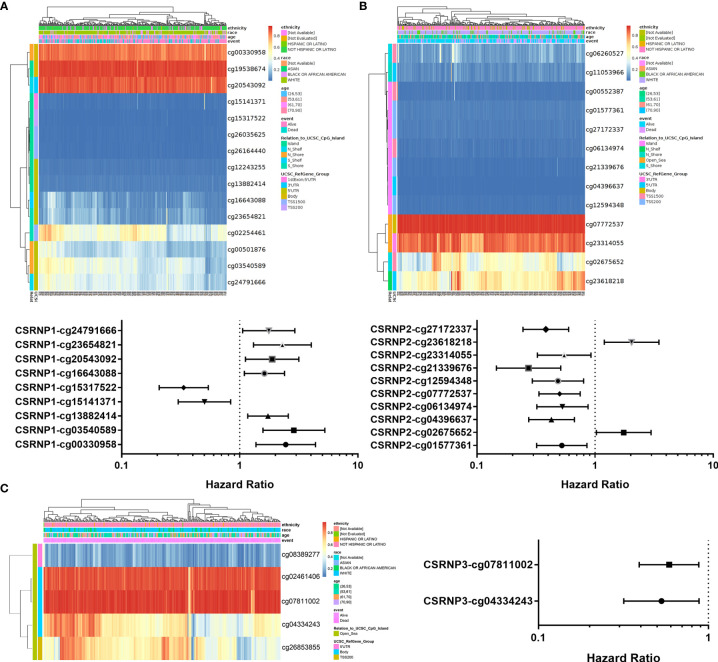
DNA methylation analysis of CSRNP family members using MethSurv. The DNA methylation clustered expression and forest plot of *CSRNP1* **(A)**, *CSRNP2* **(B)**, and *CSRNP3* **(C)**. Red to blue scale indicates high to low expression. Various colorful side boxes were used to characterize the ethnicity, race, age, event, and relation to UCSC_CpG_island and UCSC_refGene_Group.

DNA methylation also plays a pivotal role in the regulation of gene expression and affects clinical outcomes. The DNA methylation sites of the *CSRNP* genes and the prognostic values of each CpG obtained from TCGA database were analyzed by MethSurv (**Figures 6A–C** and **Table 3**). We found that cg19538674 of CSRNP1, cg07772537 of CSRNP2, and cg07811002 of CSRNP3 were the most methylated sites (**Figures 6A–C**). However, cg03540589 (HR: 2.87, 95% CI: 1.571-5.243, *P* < 0.001), cg23618218 (HR: 2.037, 95% CI: 1.196-3.469, *P* = 0.009), and cg07811002 (HR: 0.588, 95% CI: 0.392-0.879, *P* = 0.010) of *CSRNP1*, *CSRNP2*, and *CSRNP3*, respectively, were the most powerful and DNA methylation locational risk factors. Overall, nine, ten, and two CpGs of *CSRNP1*, *CSRNP2*, and *CSRNP3*, respectively, indicated aberrant prognosis (**Figures 6A–C**).

**Table 3:** The significantly prognostic values of CpG in the CSRNP family.

Gene-CpG	HR	95% CI	*P* value
CSRNP1-Body-N_Shore-cg00330958	2.448	1.369-4.377	0.002525
CSRNP1-5'UTR-N_Shore-cg03540589	2.87	1.571-5.243	0.000606
CSRNP1-5'UTR-Island-cg13882414	1.732	1.167-2.571	0.006379
CSRNP1-1stExon;5'UTR-Island-cg15141371	0.503	0.301-0.838	0.008363
CSRNP1-TSS200-Island-cg15317522	0.335	0.208-0.542	8.07E-06
CSRNP1-5'UTR-S_Shelf-cg16643088	1.615	1.092-2.388	0.016263
CSRNP1-3'UTR-N_Shelf-cg20543092	1.88	1.118-3.161	0.017233
CSRNP1-5'UTR-S_Shore-cg23654821	2.294	1.306-4.03	0.003876
CSRNP1-5'UTR-N_Shelf-cg24791666	1.759	1.057-2.926	0.029723
CSRNP2-TSS200-Island-cg01577361	0.523	0.32-0.854	0.009528
CSRNP2-TSS1500-S_Shore-cg02675652	1.747	1.026-2.977	0.040103
CSRNP2-5'UTR-Island-cg04396637	0.427	0.274-0.668	0.000188
CSRNP2-TSS1500-Island-cg06134974	0.529	0.321-0.872	0.012577
CSRNP2-Body-Open_Sea-cg07772537	0.501	0.335-0.748	0.000728
CSRNP2-TSS200-Island-cg12594348	0.485	0.294-0.8	0.004652
CSRNP2-TSS200-Island-cg21339676	0.273	0.146-0.512	5.18E-05
CSRNP2-3'UTR-Open_Sea-cg23314055	0.548	0.325-0.922	0.023602
CSRNP2-5'UTR-N_Shelf-cg23618218	2.037	1.196-3.469	0.008819
CSRNP2-TSS200-Island-cg27172337	0.383	0.245-0.597	2.32E-05
CSRNP3-Body-Open_Sea-cg04334243	0.53	0.318-0.882	0.014682
CSRNP3-Body-Open_Sea-cg07811002	0.588	0.392-0.879	0.009772

## Discussion

With the rapid development of bioinformatics tools for analyzing multiple databases with many clinical samples, outcomes, and different clinical features, prognoses can be predicted and specific cancers can be detected using biomarker molecules, especially some gene families. This study mainly explored the prognostic value and biology of *CSRNP* family genes in ccRCC using online bioinformatics tools.

*CSRNP1* has been considered as an immediate early gene (20) that binds the specific sequence AGAGTG and contains domains rich in cysteine and serine. The results of single, double, or triple gene knockouts *in vivo* indicated that the expression of *CSRNP1* could be highly induced by IL-2 in mouse T lymphocytes (6). In this study, we found that *CSRNP1* expression is positively associated with the infiltration of type 2 T helper cells in both normal and ccRCC tissues, confirming the previous findings. Noteworthy, in *Drosophila*, upregulated *CSRNP1* disturbs cell cycle progression by downregulating Cdk1 activity and promoting apoptosis in a JNK-dependent manner (21). Besides, AXUD1 (CSRNP1) upregulates cytokine-increased MMP1 expression in the articular chondrocytes (22). These findings might facilitate our understanding of the role of CSRNP1 in the progression of various types of cancers. However, whether it affects the prognosis of patients with ccRCC requires further investigation. In this study, we found that CSRNP1 could be an important suppressive prognostic factor. Decreased mRNA expression of *CSRNP1* was associated with a poor prognosis in patients with ccRCC; whereas stage-specific expression profiles significantly differed. Moreover, in terms of potential genetic and epidemic alterations, CSRNP1 acts as a protective factor in patients with high- and low-mutant burdens. In addition, nine CpGs of *CSRNP1* were correlated with a significantly aberrant prognosis.

CSRNP2 has been positively associated with many aberrant non-cancerous diseases, including obesity and type 2 diabetes mellitus (23). Moreover, Vargas et al. (24) reported that CSRNP2 acts as a potential drug repositioning candidate for the treatment of Alzheimer’s disease. However, the present study found that CSRNP2 did not sufficiently correlated with the OS of patients with ccRCC to serve as an important prognostic factor, according to the GEPIA analysis results. Furthermore, CSRNP2 was a remarkable hazard factor for patients with a high-, but not with a low-, mutant burden. In addition, the DNA methylation sites of *CSRNP2* showed significant hazard ratios, suggested that *CSRNP2* might be a meaningful target gene for epigenetic therapy.

*CSRNP3* was found to encode a transcriptional factor for muscle development in growing pigs (25), and was reported as a target gene to treat obesity and metabolic syndrome in an exome-wide mediated study (26). However, the role of CSRNP3 in cancer development requires further investigation. We found that mRNA expression of *CSRNP3*, like that of *CSRNP1*, was lower in ccRCC, and was associated with a poor prognosis. Moreover, CSRNP3 may be a protective factor in patients with high- and low-mutant burdens. In addition, two CpGs of *CSRNP3* positively correlated with significantly aberrant prognosis, which might help clarify detailed biological functions.

The prognostic values of *CSRNP* genes were consistent with the above details. We constructed a novel risk score model based on the expression of the CSRNP family to improve the prediction of OS. We also classified all the samples into high- and low-risk groups according to the median cutoff value of the risk score. The expression profiles of the CSRNP family members were different between these groups, especially those of *CSRNP1* and *CSRNP3*. The low-risk group had a better OS. Importantly, the AUC of the time-dependent ROC curve reached 0.69 over time. Moreover, this signature was an independent predictor of prognosis among patients with ccRCC. Our model exhibited good diagnostic and predictive capacities, but further improvement is needed. The CSRNP family, particularly CSRNP1 and CSRNP3, was validated as a useful prognostic biomarker for patients with ccRCC.

Further investigation on functional enrichment analysis implied that the CSRNP family might function *via* immune-related biological pathways. We found that immunocyte infiltration was higher in tumor than in paracancerous tissues. The immune infiltration profile of the CSRNP family genes in ccRCC tumor tissues was different from that in normal tissues; natural killer cells and plasmacytoid dendritic cells showed positive correlations. It is known that natural killer cells destroy various cancer cells (27–29), including renal cell carcinoma (30). Plasmacytoid dendritic cell (pDC) infiltration predicts better survival in triple-negative breast cancer (31) and melanoma (32). In addition, effector memory CD4 T cells could be considered as a protective factor in HIV (33) and cytomegalovirus disease (34). Consistently, we found that CSRNP2 and CSRNP3 were both mostly positively associated with effector memory CD4 T cells in normal tissues. Taken together, the CSRNP family might play an important role in ccRCC immune infiltration and impact the immune environment of ccRCC through immunocyte infiltration.

There were some limitations to this study. The most important was that we generated conclusions mostly based on online integrative bioinformatics analysis tools; therefore, data from *in vitro* or *in vivo* experiments, and clinical validation are urgently needed. Limitations are also imposed by the retrospective design of the study and the small sample size. Therefore, we plan to cooperate with several urological centers to conduct a prospective study and maximize the sample size. We will also continue to conduct in-depth investigations into the occurrence and development of CSRNP family genes in ccRCC to support our conclusion that the CSRNP family could serve as a useful prognostic biomarker.

In conclusion, we comprehensively explored the prognostic value of the CSRNP family using online integrative bioinformatics analysis tools. The CSRNP family signature may serve as a prognostic biomarker to predict the OS of patients with ccRCC. The risk score model based on the CSRNP showed good diagnostic and independent predictive capacity. The association between the CSRNP family and immune infiltration might offer another clinical treatment option.

## Data Availability Statement

The original contributions presented in the study are included in the article/**Supplementary Material**. Further inquiries can be directed to the corresponding author.

## Author Contributions

GY designed the study. HZ and XQ performed the bioinformatics analyses and wrote the manuscript. All authors contributed to the article and approved the submitted version.

## Funding

This study was supported by the Science and Technology Planning Project of Guangzhou City, Guangdong, China, under grant 201904010035, and the Natural Science Foundation of Guangdong Province, China, under grant 2018A030313905.

## Conflict of Interest

The authors declare that the research was conducted in the absence of any commercial or financial relationships that could be construed as a potential conflict of interest.

## Supplementary Material

Supplementary Figure 1 Expression of *CSRNP*s in patients with different Fuhrman grade ccRCCs.

## References

[1] Dunnick NR. Renal cell carcinoma: staging and surveillance. Abdominal Radiol (NY) (2016) 41:1079–85. 10.1007/s00261-016-0692-0 26971340

[2] Siegel RL Miller KD. Cancer statistics, 2020. CA Cancer J Clin (2020) 70:7–30. 10.3322/caac.21590 31912902

[3] Ferlay J Soerjomataram I Dikshit R Eser S Mathers C Rebelo M. Cancer incidence and mortality worldwide: sources, methods and major patterns in GLOBOCAN 2012. Int J Cancer (2015) 136:E359–86. 10.1002/ijc.29210 25220842

[4] Kroeger N Seligson DB Signoretti S Yu H Magyar CE Huang J. Poor prognosis and advanced clinicopathological features of clear cell renal cell carcinoma (ccRCC) are associated with cytoplasmic subcellular localisation of Hypoxia inducible factor-2alpha. Eur J Cancer (2014) 50:1531–40. 10.1016/j.ejca.2014.01.031 24565854

[5] Selvi I Demirci U. The prognostic effect of immunoscore in patients with clear cell renal cell carcinoma: preliminary results. Int Urol Nephrol (2020) 52:21–34. 10.1007/s11255-019-02285-0 31541404

[6] Gingras S Pelletier S Boyd K Ihle JN. Characterization of a family of novel cysteine- serine-rich nuclear proteins (CSRNP). PLoS One (2007) 2:e808–8. 10.1371/journal.pone.0000808 PMC1950078 17726538

[7] Espina J Feijóo CG Solís C Glavic A. csrnp1a is necessary for the development of primitive hematopoiesis progenitors in zebrafish. PLoS One (2013) 8:e53858. 10.1371/journal.pone.0053858 23326522 PMC3541188

[8] Feijóo CG Sarrazin AF Allende ML Glavic A. Cystein-serine-rich nuclear protein 1, Axud1/Csrnp1, is essential for cephalic neural progenitor proliferation and survival in zebrafish. Dev Dyn (2009) 238:2034–43. 10.1002/dvdy.22006 19544579

[9] Ishiguro H Tsunoda T Tanaka T Fujii Y Nakamura Y Furukawa Y. Identification of AXUD1, a novel human gene induced by AXIN1 and its reduced expression in human carcinomas of the lung, liver, colon and kidney. Oncogene (2001) 20:5062–66. 10.1038/sj.onc.1204603 11526492

[10] Yang HL Cho EY Han KH Kim H Kim SJ. Characterization of a novel mouse brain gene (mbu-1) identified by digital differential display. Gene (2007) 395:144–50. 10.1016/j.gene.2007.03.005 17433858

[11] Kim B Kang S Kim SJ. Differential promoter methylation and histone modification contribute to the brain specific expression of the mouse Mbu-1 gene. Mol Cells (2012) 34:433–37. 10.1007/s10059-012-0182-3 PMC3887793 23076708

[12] Tang Z Li C Kang B Gao G Li C Zhang Z. GEPIA: a web server for cancer and normal gene expression profiling and interactive analyses. Nucleic Acids Res (2017) 45:W98–102. 10.1093/nar/gkx247 28407145 PMC5570223

[13] Aguirre-Gamboa R Gomez-Rueda H Martínez-Ledesma E Martínez-Torteya A Chacolla-Huaringa R Rodriguez-Barrientos A. SurvExpress: an online biomarker validation tool and database for cancer gene expression data using survival analysis. PLoS One (2013) 8:e7425. 10.1371/journal.pone.0074250 PMC3774754 24066126

[14] Zhou Y Zhou B Pache L Chang M. Metascape provides a biologist-oriented resource for the analysis of systems-level datasets. Nat Commun (2019) 10:1523. 10.1038/s41467-019-09234-6 30944313 PMC6447622

[15] Yoshihara K Shahmoradgoli M Martínez E Vegesna R Kim H Torres-Garcia W. Inferring tumour purity and stromal and immune cell admixture from expression data. Nat Commun (2013) 4:2612. 10.1038/ncomms3612 24113773 PMC3826632

[16] Li Y Gong Y Ning X Peng D Liu L He S. Downregulation of CLDN7 due to promoter hypermethylation is associated with human clear cell renal cell carcinoma progression and poor prognosis. J J Exp Clin Cancer Res (2018) 37:276. 10.1186/s13046-018-0924-y PMC6234584 30428910

[17] Nam HY Chandrashekar DS Kundu A Shelar S Kho EY Sonpavde G. Integrative Epigenetic and Gene Expression Analysis of Renal Tumor Progression to Metastasis. Mol Cancer Res MCR (2019) 17:84–96. 10.1158/1541-7786.MCR-17-0636 30131446 PMC7222224

[18] Gao J Aksoy BA Dogrusoz U Dresdner G Gross B Sumer SO. Integrative analysis of complex cancer genomics and clinical profiles using the cBioPortal. Sci Signal (2013) 6:pl. 10.1126/scisignal.2004088 PMC4160307 23550210

[19] Modhukur V Iljasenko T Metsalu T Lokk K Laisk-Podar T Vilo J. MethSurv: a web tool to perform multivariable survival analysis using DNA methylation data. Epigenomics (2018) 10:277–88. 10.2217/epi-2017-0118 29264942

[20] Hutton JJ Jegga AG Kong S Gupta A Ebert C Williams S. Microarray and comparative genomics-based identification of genes and gene regulatory regions of the mouse immune system. BMC Genomics (2004) 5:82. 10.1186/1471-2164-5-82 15504237 PMC534115

[21] Glavic A Molnar C Cotoras D de Celis JF. Drosophila Axud1 is involved in the control of proliferation and displays pro-apoptotic activity. Mech Dev (2009) 126:184–97. 10.1016/j.mod.2008.11.005 19084594

[22] Macdonald CD Falconer AMD Chan CM Wilkinson DJ Skelton A Reynard L. Cytokine-induced cysteine- serine-rich nuclear protein-1 (CSRNP1) selectively contributes to MMP1 expression in human chondrocytes. PLoS One (2018) 13:e0207240. 10.1371/journal.pone.0207240 30440036 PMC6237337

[23] Chen J Meng Y Zhou J Zhuo M Ling F Zhang Y. Identifying candidate genes for Type 2 Diabetes Mellitus and obesity through gene expression profiling in multiple tissues or cells. J Diabetes Res (2013) 2013:970435. 10.1155/2013/970435 24455749 PMC3888709

[24] Vargas DM De Bastiani MA Zimmer ER Klamt F. Alzheimer’s disease master regulators analysis: search for potential molecular targets and drug repositioning candidates. Alzheimers Res Ther (2018) 10:59. 10.1186/s13195-018-0394-7 29935546 PMC6015462

[25] Messad F Louveau I Koffi B Gilbert H Gondret F. Investigation of muscle transcriptomes using gradient boosting machine learning identifies molecular predictors of feed efficiency in growing pigs. BMC Genomics (2019) 20:659. 10.1186/s12864-019-6010-9 31419934 PMC6697907

[26] Yamada Y Sakuma J Takeuchi I Yasukochi Y Kato K Oguri M. Identification of rs7350481 at chromosome 11q23.3 as a novel susceptibility locus for metabolic syndrome in Japanese individuals by an exome-wide association study. Oncotarget (2017) 8:39296–308. 10.18632/oncotarget.16945 PMC5503614 28445147

[27] Jo H Cha B Kim H Brito S Kwak BM Kim ST. α-Pinene Enhances the Anticancer Activity of Natural Killer Cells via ERK/AKT Pathway. Int J Mol Sci (2021) 22:656. 10.3390/ijms22020656 PMC7826552 33440866

[28] Zanker DJ Owen KL Baschuk N Spurling AJ Parker BS. Loss of type I IFN responsiveness impairs natural killer cell antitumor activity in breast cancer. Cancer Immunol Immunother (2021). 10.1007/s00262-021-02857-z PMC10992771 33449132

[29] Shi M Li ZY Zhang LM Wu XY Xiang SH Wang YG. Hsa_circ_0007456 regulates the natural killer cell-mediated cytotoxicity toward hepatocellular carcinoma via the miR-6852-3p/ICAM-1 axis. Cell Death Dis (2021) 12:94. 10.1038/s41419-020-03334-8 33462208 PMC7814008

[30] Shaffer TM Aalipour A Schürch CM Gambhir SS. PET Imaging of the Natural Killer Cell Activation Receptor NKp30. J Nucl Med (2020) 61:1348–54. 10.2967/jnumed.119.233163 PMC7456168 32532927

[31] Oshi M Newman S Tokumaru Y Yan L Matsuyama R Kalinski P. Plasmacytoid Dendritic Cell (pDC) Infiltration Correlate with Tumor Infiltrating Lymphocytes, Cancer Immunity, and Better Survival in Triple Negative Breast Cancer (TNBC) More Strongly than Conventional Dendritic Cell (cDC). Cancers (Basel) (2020) 12:3342. 10.3390/cancers12113342 PMC7697894 33198125

[32] Zhang W Lim SM Wang JH Ramalingam S Kim M Jin JO. Monophosphoryl lipid A-induced activation of plasmacytoid dendritic cells enhances the anti-cancer effects of anti-PD-L1 antibodies. Cancer Immunol Immunother (2020) 70:689–700. 10.1007/s00262-020-02715-4 32902663 PMC10991191

[33] Potter SJ Lacabaratz C Lambotte O Perez-Patrigeon S Vingert B Sinet M. Preserved central memory and activated effector memory CD4+ T-cell subsets in human immunodeficiency virus controllers: an ANRS EP36 study. J Virol (2007) 81:13904–15. 10.1128/jvi.01401-07 PMC2168869 17928341

[34] Gamadia LE Remmerswaal EB Weel JF Bemelman F Van Lier RA Ten Berge IJ. Primary immune responses to human CMV: a critical role for IFN-gamma-producing CD4+ T cells in protection against CMV disease. Blood (2003) 101(7):2686–92. 10.1182/blood-2002-08-2502 12411292

