# Supplementary figures and images for: The CSRNP Gene Family Serves as a Prognostic Biomarker in Clear Cell Renal Cell Carcinoma
Source: Front Oncol. 2021 Mar 31;11:620126. doi: 10.3389/fonc.2021.620126 (PMC8045970; doi:10.3389/fonc.2021.620126)

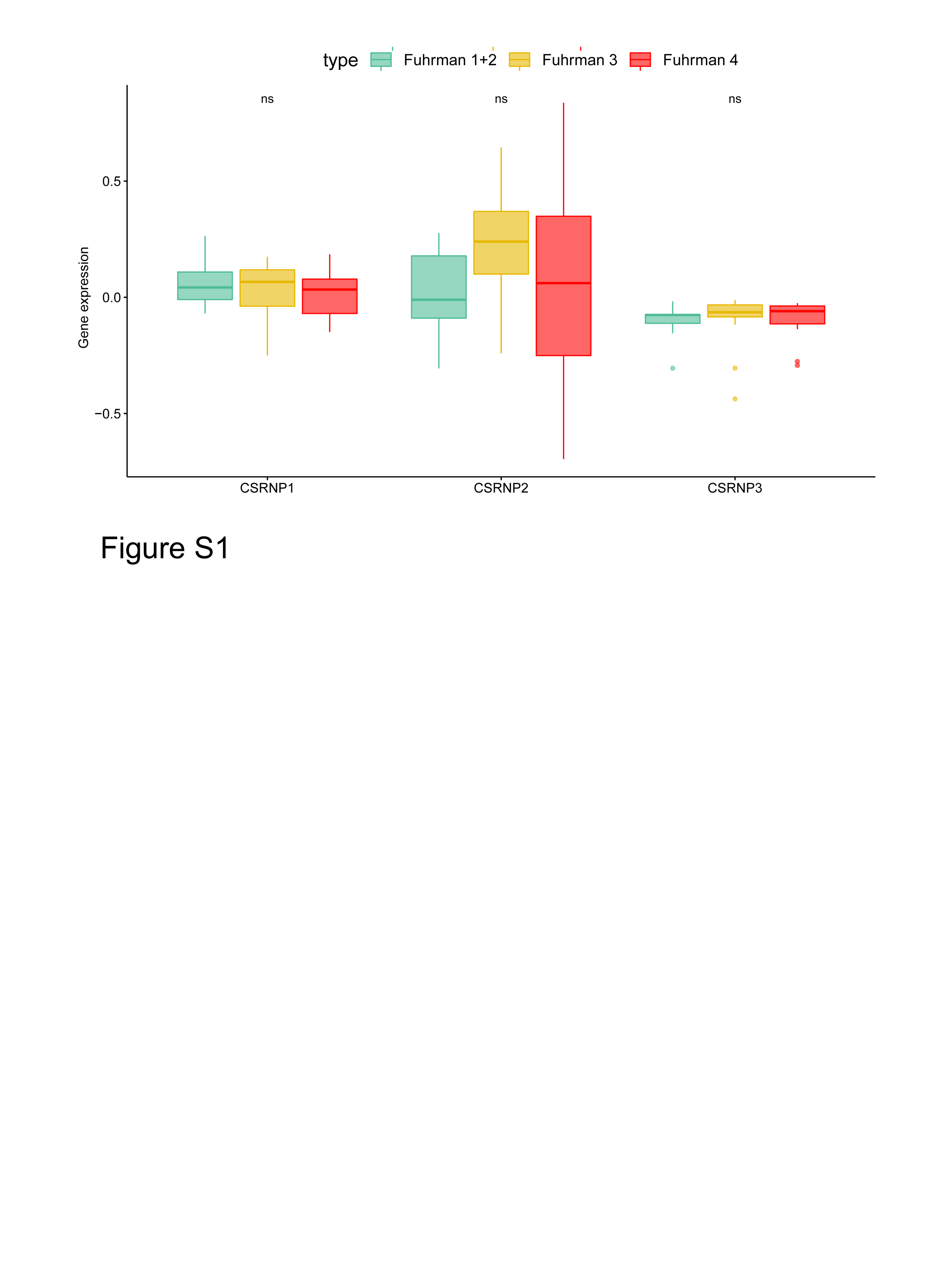

Supplement: Supplementary Figure 1 — Expression of CSRNPs in patients with different Fuhrman grade ccRCCs. [file Image_1.tif]
